# Advanced antireflection for back-illuminated silicon photomultipliers to detect faint light

**DOI:** 10.1038/s41598-022-18280-y

**Published:** 2022-08-16

**Authors:** Yuguo Tao, Arith Rajapakse, Anna Erickson

**Affiliations:** Nuclear and Radiological Engineering, Georgia Institute of Technology, Atlanta, GA USA

**Keywords:** Electrical and electronic engineering, Materials for devices, Materials for optics, Optical physics, Electronics, photonics and device physics, Engineering, Materials science, Optics and photonics, Physics

## Abstract

Silicon photomultipliers have attracted increasing attention for detecting low-density light in both scientific research and practical applications in recent years; yet the photon losses due to reflection on the light-sensitive planar silicon surface considerably limit its photon detection efficiency. Here we demonstrate an advanced light trapping feature by developing the multi-layer antireflection coatings and the textured silicon surface with upright random nano-micro pyramids, which significantly reduces the reflection of faint light in a wide spectrum, from ultraviolet to infrared. Integrating this advanced photon confinement feature into next-generation back-illuminated silicon photomultiplier would increase the photon detection efficiency with significantly lower reflection and much more active areas. This advanced design feature offers the back-illuminated silicon photomultiplier broader application opportunities exemplified in the emerging scenarios such as nuclear medical imaging, light detection and ranging for autonomous driving, detection of scintillation light in ionizing radiation, as well as high energy physics.

## Introduction

Silicon photomultipliers (SiPM) are the cornerstones of photodetector technologies for detecting faint light in both industry and scientific research since their early development in 1990s^1–5^. Their attractive performances have brought benefits into emerging applications, such as ionizing radiation detection, biomedical imaging, and light detection and ranging (LiDAR) for autonomous driving^6^. Compared to photomultiplier tube (PMT) that is a conventional photodetector legacy technology in the radiation detection, SiPM offers several advantages, including low operation voltage, compactness, ruggedness, and relatively low cost^7^. Furthermore, in contrast to PMT, SiPM is insensitive to magnetic fields, which leads to its vital role as the foundation of light detection technology for the advanced medical equipment in the presence of magnetic fields, such as positron emission tomography (PET) imaging. However, the photon detection efficiency (*PDE*) that is defined as the ratio between the numbers of detected photons and the photons arriving at the detector, which is also one of the key measurable metrics that quantify SiPM’s performance, is still limited to about 60% and rapidly decreases from the peak as the wavelength enters into the ultraviolet range^5,8^. This is due to not only the reflection losses of photons impinging on the front planar silicon surface, but also the limited fill factor (*FF*) caused by the dead areas (*i.e.*, quenching resistor, isolation trench, guard ring, and contact metal) for the conventional front-illuminated structure, in addition to the recombination loss of the photo-generated primary carriers near defect centers^9^. Therefore, a reduction in photon reflection at the surface is a prominent role in the development of high-performance SiPM devices.

In order to reduce the photon losses due to reflection, the approach of antireflection coatings (ARC) is typically used, because it can reduce the photon losses by making use of phase changes and the dependence of the reflectivity on refractive index^10^. In addition to the proper refractive index and film thickness, a low extinction coefficient (*κ*) is also required for the ARC material to avoid a significant photon absorption by the ARC thin-film layer. The ARC materials used in conventional SiPM are thermally grown silicon dioxide (SiO_2_), and silicon nitride (SiN_x_) that can be deposited by plasma-enhanced chemical vapor deposition (PECVD) as single-layer ARC (SARC) on planar surface^2,7,11^. In this work, we developed multi-layer ARC on textured surface with upright nano-micro pyramids to reduce the reflection, including double-layer ARC (DARC) and triple-layer ARC (TARC). For comparison purpose, the single-layer ARC as well as the bare silicon wafer without ARC on both planar and textured surfaces are also studied in this work. In the end, the back-illuminated SiPM integrated with the multi-layer ARC on textured surface are discussed, as a comparison to the conventional SiPM with ARC on planar surface.

## Results

### Conventional single-layer antireflection coating

**Figure 1:**
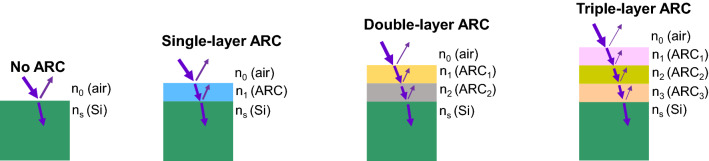
Schematic of various ARC features on silicon wafers, including no ARC, single-layer ARC, double-layer ARC, and triple-layer ARC. Refractive indices of different layers are indicated.

According to the theory of quarter-wave dielectric layer^12,13^, the reflection of incident beam of light reflected from the surface of a single-layer ARC deposited on a substrate material has its minimum value when:

1 $$ n_{1} = \sqrt {n_{0} n_{s} } $$

2 $$ n_{1} d_{1} = \lambda_{0} /4 $$

where *n*_*0*_, *n*_*1*_, and *n*_*s*_ represent the refractive indices of medium (air in this work), ARC material, and absorption substrate material (silicon in this work), respectively. *d*_*1*_ is the thickness of ARC layer, and *λ*_*0*_ is the wavelength of incident light with minimum reflection. For a SiPM in air (*n*_*0*_ = 1.0, and *n*_*s*_ = 3.8), the optimum refractive index of single-layer ARC is *n*_*1*_ ≈ 1.9. Note that the specific refractive index values given in this work are measured at the wavelength of 632 nm. Therefore, this work applied the conventional single-layer ARC material SiNx as a reference due to its refractive index of 1.96 close to the optimum value, and the SiO_2_ was also included as a comparison because it is typically used as a surfa passivation material as well as an ARC material at the same time for the early SiPM studies^2,7^. Figure 1 shows the schematic of single-layer ARC on silicon wafers in this study, the case of no ARis also included.

**Figure 2:**
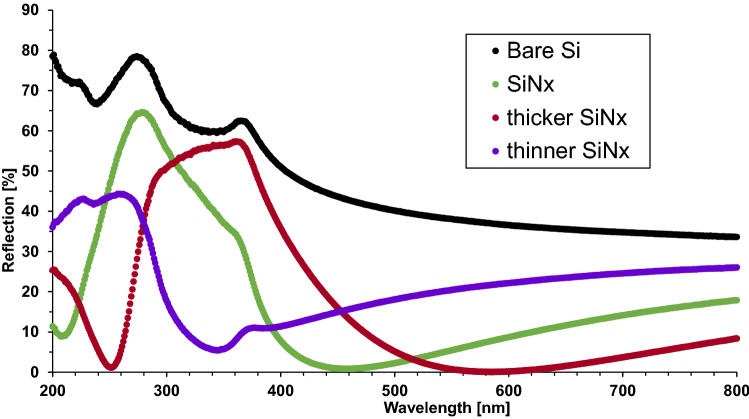
Reflection comparison of SiN_x_ (55 nm), “thicker” SiN_x_ (72 nm), and “thinner” SiN_x_ (38 nm) as single-layer ARC deposited on the silicon wafers with planar silicon surface. The case of without any ARC (“Bare Si”) is also concluded as a reference.

Figure 2 shows that the 55-nm SiN_x_ deposited on planar silicon surface as a single-layer ARC can minimize the reflection close to zero at the wavelength of 450 nm, which is in good agreement with the condition Eqs. (1)–(2). The “thicker” SiN_x_ (72 nm) shifts the reflection curve to higher wavelength with the minimum reflection close to zero at around 600 nm, while the “thinner” SiN_x_ (38 nm) results in the minimum reflection of still over 5% at about 340 nm, suggesting the thickness of 38 nm is too thin for SiN_x_ as a single-layer ARC. It is also indicated in Fig. 2 that the average reflection of all studied SiN_x_ films as single-layer ARC measured in the spectrum of 200–800 nm are all over 15%, although the minimum reflection can be reduced to zero at certain wavelength by changing the SiN_x_ thickness.

**Figure 3:**
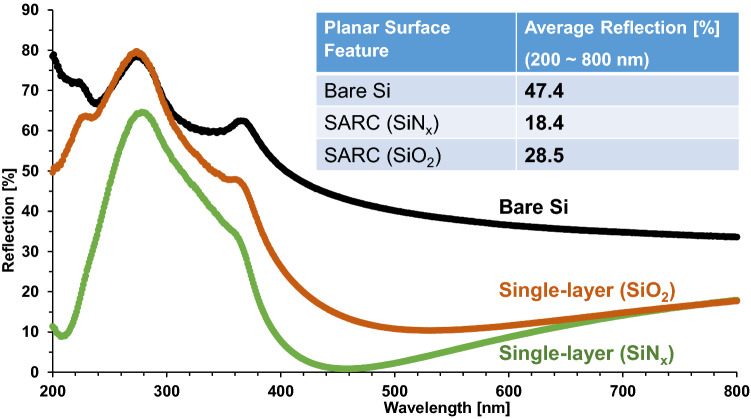
Reflection comparison of SiN_x_ and SiO_2_ as single-layer ARC on planar silicon surface. The insert table lists the average reflection from the measurement results.

Although SiO_2_ demonstrates a better surface passivation function than SiN_x_ due to lower interface defect density^14^, and the earlier SiPM studies use it as a surface passivation layer as well as a single-layer ARC^2^, Fig. 3 clearly shows that SiO_2_ has inferior antireflection performance over the studied spectrum. Compared to the case of without any ARC (bare silicon surface), SiO_2_ does reduce the reflection shown in Fig. 3, and the average reflection is reduced from 47.4% to 28.5%. However, it is still much higher than that of SiN_x_ (18.4%), as summarized by the insert table in Fig. 3. Therefore, Fig. 3 reveals that SiO_2_ is not a proper ARC material, and SiN_x_ as a single-layer ARC is still not sufficient to reduce photon losses for high-performance next-generation SiPM devices.

### Multi-layer ARC: double-layer ARC and triple-layer ARC

Although a single-layer ARC is able to minimize the reflection down to zero at a specific wavelength when it meets the conditions as given by Eqs. (1) and (2), its reflections at other wavelengths are still high because its refractive index is wavelength-dependent. Therefore, to reduce the reflection over a wide spectrum of 200 ~ 800 nm, this work developed multi-layer ARC, including double-layer ARC and triple-layer ARC, as shown in Fig. 1. The multi-layer ARC feature can decrease the reflection at the surface because of the interference of the reflected light from each interface. This work applied the transfer matrix method (TMM)^10,15^ in addition to the theory of quarter-wave dielectric layers to determine the multi-layer ARC materials and their thickness.

For the double-layer ARC feature, to minimize the average reflection over a wide spectrum, the selected ARC materials should meet the conditions:

3 $$ n_{1} = \sqrt[3]{{n_{0}^{2} \cdot n_{s} }} $$

4 $$ n_{2} = \sqrt[3]{{n_{0} \cdot n_{s}^{2} }} $$

5 $$ d_{j} = \frac{{\lambda_{0} }}{{4n_{j} }} $$

where *n*_*0*_, *n*_*1*_, *n*_*2*_, and *n*_*s*_ represent the refractive dices of each layer as shown in Fig. 1, and *d*_*j*_ (*j* = 1, and 2) is the thickness of ARC layer. Therefore, according to the conditions (3) and (4), magnesium fluoride (MgF_2_, *n*_*1*_ = 1.37) and zinc sulfide (ZnS, *n*_*2*_ = 2.33) are selected as constituent layers of the double-layer ARC feature in this work.

Similarly, for the triple-layer ARC feature, the following conditions of refractive indices of the selected ARC materials should be met to minimize the average reflection over a wide spectrum:

6 $$ n_{1} = \sqrt[4]{{n_{0}^{3} \cdot n_{s} }} $$

7 $$ n_{2} = \sqrt {n_{0} \cdot n_{s} } $$

8 $$ n_{3} = \sqrt[4]{{n_{0} \cdot n_{s}^{3} }} $$

where *n*_*0*_, *n*_*1*_, *n*_*2*_, *n*_*3*_, and *n*_*s*_ are the refractive indices of each layer as shown in Fig. 1. The thickness of each layer *d*_*j*_ (*j* = 1, 2, and 3) can be determined by Eq. (5). So, this work investigated MgF_2_ (*n*_*1*_ = 1.37), HfO_2_ (*n*_*2*_ = 1.91), and TiO_2_ (*n*_*3*_ = 2.49) for the triple-layer ARC feature.

**Figure 4:**
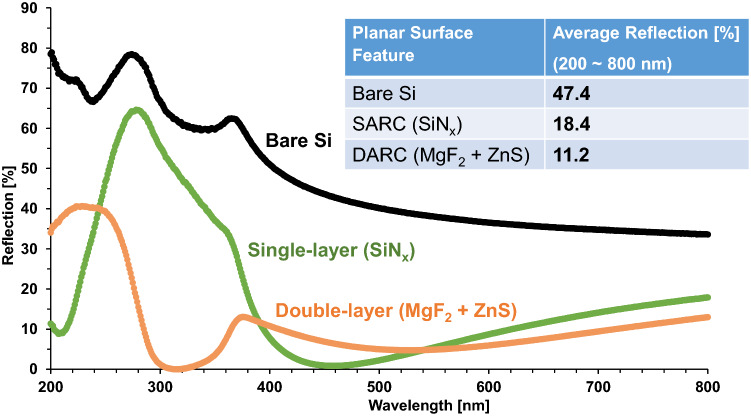
Reflection comparison of SiN_x_ as single-layer ARC (SARC) and MgF_2_/ZnS as a double-layer ARC (DARC) on planar silicon surface. The insert table lists the average reflection obtained from the measurement results.

Figure 4 shows that double-layer ARC reduces the reflection for most wavelengths, compared to SiN_x_ as single-layer ARC, especially in the ultraviolet (UV) range, with the minimum reflection close to zero at round 320 nm. Its resulted average reflection also decreases to 11.2%. However, its reflection at wavelength below 280 nm is still high (over 15%), and even higher than that of SiN_x_ at below 250 nm. Therefore, this work develops the textured surface in combination with multi-layer ARC features to enhance the light trapping and hence reduce the photon losses.

### Textured surface with upright random nano-micro pyramids formed by anisotropic etching

**Figure 5:**
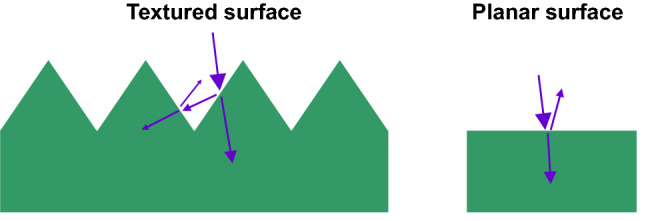
Schematic comparison of the reflected light on textured and planar silicon surfaces.

To enhance the light trapping on silicon wafer, texturing the wafer surface by an anisotropic etching to obtain upright random nano-micro pyramidal structures^16,17^ is an effective approach to reduce the surface reflection. As shown Fig. 5, compared to the planar surface where the light reflected from the surface is completely lost, the textured surface can allow the light reflected from the side of one of pyramids to be reflected downward, and then getting a second chance of being absorbed into the silicon bulk. The formation of these random arrays of ideal {111} faceted upright pyramids on the etched surface is driven by the anisotropic nature of the chemical etchant, that is, the etch rate in the < 100 > direction of single crystalline silicon is several times greater than that in the < 111 > direction. The higher etch rate in the < 100 > direction is because the {100} surface requires the lower activation energy to remove an atom than the {111} surface^18^. This is because the {100} surface only needs to break two back bonds rather than three ones in the case of {111} surface^19^. Note that each atom of Si {111} surface has three back bonds and one dangling bond, but each atom of {100} surface has two back bonds and two dangling ones. Therefore, the upright pyramids are formed by the intersection of (100) and (111) planes as the consequence of anisotropic etching of silicon (100) substrate due to the slowest etching rate of {111} crystallographic planes.

**Figure 6:**
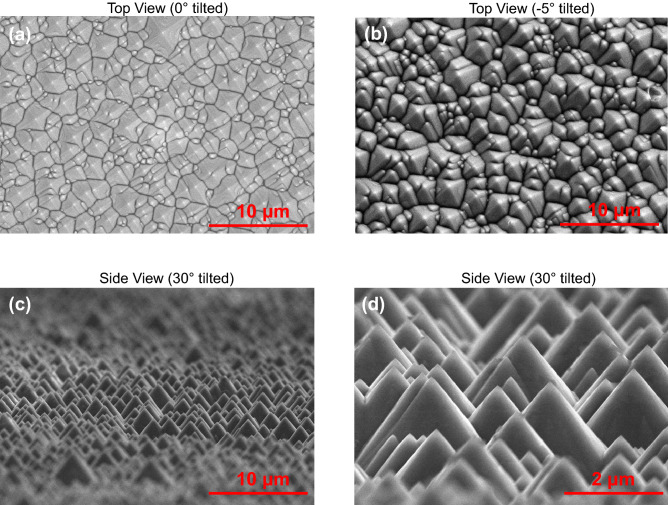
SEM images of textured silicon (100) wafer surface after the anisotropic etching: (**a**) top view, (**b**) top view with sample tilted at − 5°, (**c**) side view with sample tilted at 30°, (**d**) side view with sample tilted at 30° by high resolution.

Figure 6 displays the surface morphology of etched silicon (100) wafers by scanning electron microscope (SEM) images taken on the textured surface after the anisotropic etching. The appearance of the upright random nano-micro pyramids is clearly seen from these SEM images. The sizes of these pyramids are random, in the range of a few hundreds of nanometers to a couple of microns. These upright pyramids are formed due to the anisotropic etching of silicon (100) substrate, which are bounded by {111} crystallographic planes because of the slowest etching rate of {111} planes^18^. The image (a) is obtained from the top view, showing the pyramid tips as shining points and the pyramid base borders as black lines, while the resulted image (b) gives a more straightforward view after tilting the sample at -5°. From the side view and tilting the sample at 30°, image (c) shows an art-like overview of resulted upright pyramids, and image (d) gives a closer look after increasing the SEM resolution.

**Figure 7:**
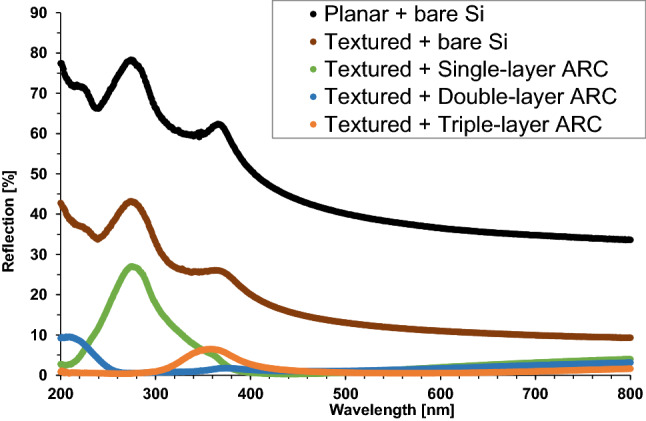
Reflection comparison of single-layer, double-layer, and triple-layer ARC on textured silicon surfaces. The bare planar and textured silicon surfaces are also included.

As shown in Fig. 7, the textured bare surface has much lower reflection than the planar bare surface over the entire spectrum, because it allows the light reflected from the side of pyramids to be reflected downward and hence get a second chance of being absorbed into silicon, as demonstrated in Fig. 5. This leads to its average reflection of only 18.6%, a dramatical decrease from that of the planar surface (47.4%). Figure 7 also indicates that the reflection reduction between the textured and planar surfaces is almost similar for all wavelengths because of the same light trapping mechanism regardless of the wavelength. To further reduce the reflection, various ARC dielectric stacks are deposited on the textured surface to combine the antireflection benefits of both the textured surface and the ARC feature.

### Multi-layer ARC on textured surface

**Figure 8:**
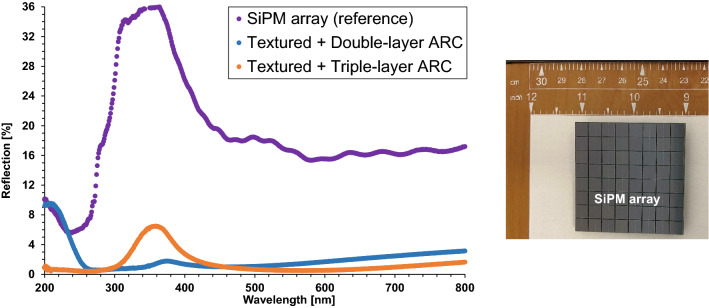
Reflection comparison of multi-layer ARC and commercial SiPM array as a reference. The photo of SiPM array is included.

It is shown in Fig. 7 and Fig. 8 that the single-layer ARC (SiN_x_) on textured surface provides a dramatical decrease in reflection, compared to the bare textured surface. Its reflection is close to zero for the wavelength above 400 nm, while it bumps up in the UV range, and peaks at over 27% around 275 nm. These result in its average reflection of 5.0% on textured surface, an effective improvement from the same ARC feature on planar surface (18.4%) shown in Fig. 3 and Fig. 4.

Having the reflection as low as the single-layer ARC for the wide spectrum from blue light to red light, the double-layer ARC (MgF_2_/ZnS) on textured surface cuts down significantly the reflection for the UV light, as shown in Fig. 7 and Fig. 8. However, its reflection gradually increases at wavelength below 260 nm, and saturates at approximately 9% around 200 nm. These lead to a very low average reflection of 2.3%, compared to 11.2% for the same double-layer ARC feature on planar surface listed in Fig. 4, a very impressive enhancement in antireflection.

**Figure 9:**
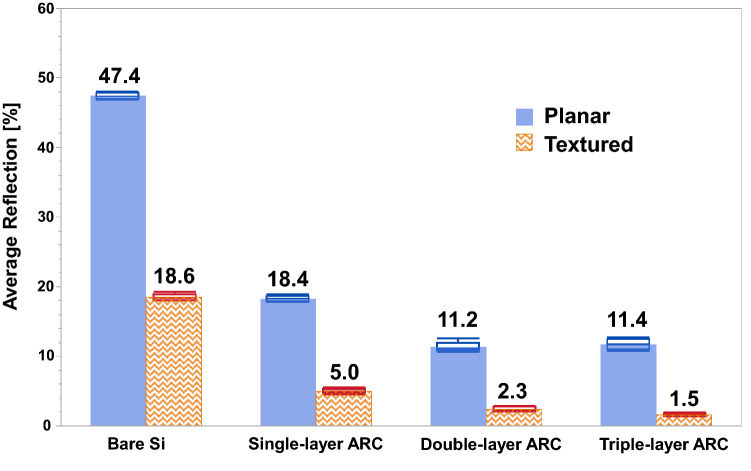
Comparison summary of the average reflections of bare Si (no ARC), single-layer, double-layer, and triple-layer ARC on planar and textured surfaces. The error bars are included.

Figure 7 and Fig. 8 display that the triple-layer ARC (MgF_2_/HfO_2_/TiO_2_) on textured surface can reduce the reflection down to less than 1% for the wavelengths below 260 nm, which is a very promising enhancement, compared to the double-layer ARC feature. Its reflection also is marginally lower at the wavelength above 500 nm than the double-layer ARC, but bumps up in the range of 300 ~ 420 nm, and peaks at 6.5% around 360 nm. These result in an average reflection of 1.5%, the lowest among the studied antireflection features in this work. Figure 9 summarizes the average reflections of single/double/triple-layer ARC on planar and textured surfaces, as well as the bare planar and textured surfaces without ARC. It clearly displays that all ARC features on textured surface have lower average reflection than their counterparts on planar surface. In addition, the multi-layer ARC features can reduce the average reflection more than the single-layer ARC regardless of the silicon wafer surface tomography, either planar or textured, as shown in Fig. 9.

When SiPM is coupled with a scintillator for radiation detection applications, the sensitive wavelength of SiPM must match with the emission wavelength of scintillators. For example, barium fluoride (BaF_2_) scintillators are among the fastest scintillators with sub-nanosecond decay time for emitted “fast” pulses at wavelength of 220 nm, and widely used in the applications of time-of-flight measurement, PET, nuclear and high energy physics^20^. Therefore, in order to detect these “fast” pulses emitted from BaF_2_ scintillators, the triple-layer ARC on textured surface would be a better feature than the double-layer ARC, because it enables lower reflection at wavelength range of 220 nm shown in Fig. 8, and hence potentially higher *PDE*. While detecting the photons with wavelength in the range of 300 ~ 420 nm, i.e., emitted from cesium fluoride (CsF), cerium fluoride (CeF_3_), and yttrium orthoaluminate (YAlO_3_) scintillators^20^, the double-layer ARC on textured surface may be a competitive option, but the triple-layer ARC on textured surface is still a better feature for detecting photons with wavelength over 460 nm, i.e., coming from Y_3_A1_5_O_12_ and CsI:Tl scintillators^21^. In the next section, we will discuss more about the back-illuminated SiPM with the multi-layer ARC on textured surface.

## Discussion

**Figure 10:**
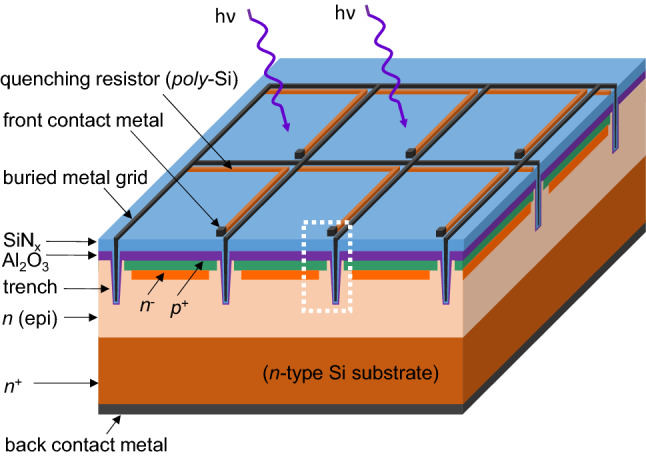
Schematic of conventional front-illuminated SiPM with SiN_x_ as single-layer ARC on planar surface. The section highlighted by the dash-line rectangle is enlarged and shown in the left part of Fig. 11.

**Figure 11:**
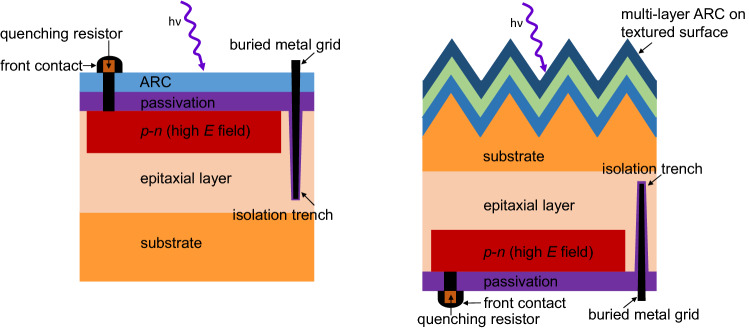
Schematic comparison of the SiPM cross-sections: (left) the conventional front-illuminated SiPM with ARC on planar surface, and (right) the back-illuminated SiPM with multi-layer ARC on textured surface that is developed in this work.

Figure 10 shows the schematic of conventional front-illuminated SiPM with SiN_x_ as single-layer ARC on planar surface where photons are incident. As a solid-state photodetector, SiPM is segmented in tiny Geiger-mode Avalanche Photodiodes (GM-APD), also called microcells. The cross-section of a microcell of this SiPM structure is enlarged and shown in Fig. 11 (left). The *pn*-junction of microcell is formed on the top of epitaxial layer that is grown on the *n*-type Si wafer substrate to create a high-field region for the Geiger-mode avalanche events via a sufficient bias voltage. The Al_2_O_3_ thin film acts as an excellent passivation layer to passivate the surface defects (i.e., dangling bonds) to suppress the carrier recombination by its low interface defect density and built-in negative fixed charges at the interface to shield the electron carriers^14^. The front contact metal is connected to the positively-doped emitter layer (*p*^+^) and the quenching resistor that is used to turn off the avalanche current to reduce the operating voltage down to below the breakdown voltage, so that the microcell can be restored to its initial status and get ready to detect a new event. Through the quenching resistors, each microcell is connected in parallel to the front buried metal grid. The isolation trenches between microcells are introduced to minimize the optical cross-talk effect that is one of noise sources, but it reduces the total active areas and hence the *FF*. In addition, the guard ring structure at the edge area with lower doping level (*p*^*-*^) than the active area (*p*^+^) can prevent an edge premature breakdown, but induces a reduction in the active area. As a consequence, this conventional front-illuminated SiPM structure has not only a high photon reflection due to the ARC on planar surface but also a limited *FF* due to the dead areas (i.e., quenching resistor, front contact mental, isolation trench, and guard ring) on the detector side where the photons are incident, which limits quantum efficiency (*QE*) and hence *PDE*. Therefore, this work investigates the back-illuminated SiPM with multi-layer ARC on textured surface.

Figure 11 compares the microcell cross-sections of the conventional front-illuminated SiPM with ARC on planar surface and the back-illuminated SiPM with multi-layer ARC on textured surface that is developed in this work. For the conventional front-illuminated SiPM, the incoming photons are incident on the planar surface coated by ARC, being on the same side as the avalanche regions (high electrical field regions). In contrast, for the back-illuminated SiPM, the incoming photons fall on the detector side opposite to the avalanche regions^22–24^. The multilayer ARC on textured surface developed in this work is designed on the detector side of back-illuminated SiPM shown in Fig. 11, directly facing the incoming photons. As shown in Fig. 8, the multi-layer ARC on textured surface has much lower reflection over the entire spectrum than the SiPM array that has the conventional front-illuminated structure with ARC on planar surface. Note that this SiPM array is SensL J-Series sensors (635J1) with microcell size of 35 × 35 μm^2^, *FF* of 75%, the maximum *PDE* of 50% at 420 nm, dark count rate of 150 kHz/mm^2^, and gain of 6.3 × 10^6^. The resulted average reflection of this SiPM array is 18.9%, much higher than those of the multi-layer ARC on textured surface, 2.3% for the double-layer ARC and 1.5% for the triple-layer ARC. So, applying the multi-layer ARC on textured surface to the back-illuminated SiPM can reduce dramatically its reflection and hence potentially boost its *PDE* and *QE* that is defined as the probability for an incident photo to generate a primary electron–hole pair and subsequent collection of photogenerated carriers in the active area of the SiPM device.

In addition, as the dead areas (including quenching resistor, isolation trench, and guard ring) are relocated to the microcell’s bottom side, the back-illuminated SiPM structure has more active areas on the top side where photons are incident, which leads to a much higher *FF*, compared to the conventional front-illuminated structure that typically limits the *FF* to 80% or even much lower for smaller microcell sizes. Combining the high *FF* (potentially close to 100%) and the low reflection as described above, the back-illuminated SiPM structure with multi-layer ARC on textured surface shown in Fig. 11 would improve the *PDE* to a very promising level.

In conclusion, this work has demonstrated that the multi-layer ARC on textured silicon surface with upright random nano-micro pyramids can dramatically reduce the reflection over a wide spectrum, compared to the conventional single-layer ARC on planar surface for SiPM. On the planar silicon surface, SiN_x_ as the conventional single-layer ARC has a better antireflection performance than SiO_2_ due to its optimum refractive index, and can minimize the reflection down close to zero at specific wavelengths for the optimum film thickness, but its average reflection of 18.4% is higher than that of the multi-layer ARC (about 11%). The textured surface can be formed by the anisotropic etching of silicon (100) substrate in alkaline solution due to the slowest etching rate of {111} crystallographic planes, and lead to significantly lower reflection than the planar surface regardless of the wavelength, resulting in the average reflection of 18.6% vs 47.4%. This is due to its powerful light trapping feature that allows the light reflected from the side of pyramids to be reflected downward and get a second chance of being coupled into silicon. The combined feature of the multi-layer ARC deposited on the textured surface empowers the light trapping performance even further by reducing the average reflection down to 2.3% for the double-layer ARC (MgF_2_/ZnS), and 1.5% for the triple-layer ARC (MgF_2_/HfO_2_/TiO_2_), which is lower than that of the single-layer ARC on textured surface (5.0%).

Compared to the conventional front-illuminated SensL SiPM array with ARC on planar surface that have an average reflection of 18.9%, a *FF* of 75%, and a state-of-the-art *PDE* of 50%, our back-illuminated SiPM with the multi-layer ARC on textured surface would increase the *PDE* to a very promising level by the ultra-low average reflection of 1.5% and the much higher *FF* (potentially approach to 100%) due to the elimination of the dead areas from the detector side. Benefiting from its reflection close to zero for the wavelength range of 200 ~ 300 nm, the studied feature of the multi-layer ARC on textured surface might enable the back-illuminated SiPM to effectively detect the “fast” pulses with sub-nanosecond decay time at wavelength of 220 nm emitted from one of the fastest scintillators BaF_2_, which paves a way to facilitate its broad applications, such as time-of-flight measurement, PET, scintillation light detection in ionizing radiation, nuclear and high energy physics.

## Methods

The silicon wafers used in this study are polished float-zone 4-inch *n*-type (100) wafers with thickness of 280 μm. The wafers were processed in a 5% hydrogen fluoride (HF) solution prior to depositing ARC thin films. The SiN_x_ dielectric thin films were deposited by PECVD at 250 °C with precursors of nitrogen, silane, and ammonia. Three different thicknesses of SiN_x_ films are compared, including a designated thickness of 55 nm in order to detect the photons emitted from conventional scintillators with wavelength around 450 nm, a “thicker” film of 72 nm, and a “thinner” one of 38 nm. The 78-nm SiO_2_ films (refractive index *n*_*SiO2*_ = 1.46) used to compare SiN_x_ in the single-layer ARC feature were also deposited by PECVD at 250 °C with precursors of nitrogen, silane, and nitrous oxide. For the multi-layer ARC features, zinc sulphide (ZnS, refractive index *n*_*ZnS*_ = 2.20) and magnesium fluoride (MgF_2_, refractive index *n*_*MgF2*_ = 1.37) thin films were grown by a thermal-evaporator at the voltages of 15 V and 10 V and the deposition rate of 2 Å/s, with the film thicknesses of 48 nm and 77 nm. Titanium oxide (TiO_2_, refractive index *n*_*TiO2*_ = 2.49) and hafnium oxide (HfO_2_, refractive index *n*_*HfO2*_ = 1.91) thin films are deposited by an E-beam evaporator at the deposition rate of 0.5 Å/s. The film thicknesses of triple-layer ARC are 42, 55, and 77 nm for TiO_2_, HfO_2_, MgF_2_, respectively. Note that the physical vapor deposition (PVD) techniques used this work are the thermal-evaporator and the E-beam evaporator, which are less conformal than the chemical vapor deposition (CVD) or the atomic-layer deposition (ALD) techniques. However, because the pyramids with feature base angle of about 54° formed on the silicon wafer surface are in the scale of one micron while the ARC thickness in this study is in the scale of 100 nm, the applied deposition technique would have negligible impact on the reflection measurement results. After the ARC depositions, the wafers were characterized by a Cary 5000 UV–Vis/NIR spectrophotometer to measure the total reflectance (specular and scattering) in the wide spectrum of 200 ~ 800 nm, which is enabled by using a combination of a tungsten halogen and deuterium arc light source to illuminate the samples.

The anisotropic etching was performed in the 3% (volume ratio) potassium hydroxide (KOH) alkaline and 4% isopropyl alcohol (IPA) and deionized (DI) water mixture solutions at a high temperature of 80 °C for 20 min. After the texturing, the wafers were cleaned in a 5% HF solution. Note that all the wet chemical processing and the thin film depositions were performed in the specialized class 100 cleanrooms to avoid contaminations. This work used the scanning electron microscope (SEM) technique at 5 kV to characterize the surface features after the texturing process.

## Data Availability

The data that support the findings of this study are available from the corresponding author on request.

## References

[1] Bisello D, Gotra Y, Jejer V, Kushpil V, Malakhov N, Paccagnella A, Sadygov Z, Stavitsky I, Tsyganov E (1995). Silicon avalanche detectors with negative feedback as detectors for high energy physics. Nucl. Instrum. Methods Phys. Res. Sect. A.

[2] Antich PP, Tsyganov EN, Malakhov NA, Sadygov ZY (1997). Avalanche photo diode with local negative feedback sensitive to UV, blue and green light. Nucl. Instrum. Methods Phys. Res. Sect. A.

[3] Buzhan P, Dolgoshein B, Filatov L, Ilyin A, Kantzerov V, Kaplin V, Karakash A, Kayumov F, Klemin S, Popova E, Smirnov S (2003). Silicon photomultiplier and its possible applications. Nucl. Instrum. Methods Phys. Res. Sect. A.

[4] Roncali E, Cherry SR (2011). Application of silicon photomultipliers to positron emission tomography. Ann. Biomed. Eng..

[5] Sadygov Z, Sadigov A, Khorev S (2020). Silicon photomultipliers: Status and prospects. Phys. Part. Nucl. Lett..

[6] Li Y, Ibanez-Guzman J (2020). Lidar for autonomous driving: The principles, challenges, and trends for automotive lidar and perception systems. IEEE Signal Process. Mag..

[7] Renker D (2006). Geiger-mode avalanche photodiodes, history, properties and problems. Nucl. Instrum. Methods Phys. Res. Sect. A.

[8] Nagai A, Alispach C, Berghöfer T, Bonanno G, Coco V, Dellavolpe D, Haungs A, Heller M, Henjes-Kunst K, Mirzoyan R, Montaruli T (2018). SENSE: A comparison of photon detection efficiency and optical crosstalk of various SiPM devices. Nuclear Instrum. Methods Phys. Res. Sect. A Accel. Spectrom. Detectors Assoc. Equip..

[9] Piemonte C, Gola A (2019). Overview on the main parameters and technology of modern Silicon Photomultipliers. Nucl. Instrum. Methods Phys. Res., Sect. A.

[10] Zhao J, Green MA (1991). Optimized antireflection coatings for high-efficiency silicon solar cells. IEEE Trans. Electron Devices.

[11] Dinu, N., 2016. Silicon photomultipliers (SiPM). In *Photodetectors* (pp. 255–294). Woodhead Publishing.

[12] Heavens, O.S., 1991. Optical properties of thin solid films. Courier Corporation.

[13] Green, M.A., 1982. Solar cells: operating principles, technology, and system applications. *Englewood Cliffs*.

[14] Tao Y, Erickson A (2022). Enhanced surface passivation by atomic layer deposited Al_2_O_3_ for ultraviolet sensitive silicon photomultipliers. IEEE Trans. Nucl. Sci..

[15] Saylan S, Milakovich T, Hadi SA, Nayfeh A, Fitzgerald EA, Dahlem MS (2015). Multilayer antireflection coating design for GaAs0. 69P0. 31/Si dual-junction solar cells. Sol. Energy.

[16] Campbell P, Green MA (1987). Light trapping properties of pyramidally textured surfaces. J. Appl. Phys..

[17] Baker-Finch SC, McIntosh KR (2013). Reflection distributions of textured monocrystalline silicon: Implications for silicon solar cells. Prog. Photovoltaics Res. Appl..

[18] Seidel H, Csepregi L, Heuberger A, Baumgärtel H (1990). Anisotropic etching of crystalline silicon in alkaline solutions: I. Orientation dependence and behavior of passivation layers. J. Electrochem. Soc..

[19] Chen W, Liu Y, Yang L, Wu J, Chen Q, Zhao Y, Wang Y, Du X (2018). Difference in anisotropic etching characteristics of alkaline and copper based acid solutions for single-crystalline Si. Sci. Rep..

[20] Blasse G (1994). Scintillator materials. Chem. Mater..

[21] Yanagida T (2018). Inorganic scintillating materials and scintillation detectors. Proc. Japan Acad. Ser. B.

[22] Otte AN, Dolgoshein B, Hose J, Klemin S, Lorenz E, Lutz G, Mirzoyan R, Popova E, Richter RH, Struder LWJ, Teshima M (2006). Prospects of using silicon photomultipliers for the astroparticle physics experiments EUSO and MAGIC. IEEE Trans. Nucl. Sci..

[23] Moser, H.G., Hass, S., Merck, C., Ninkovic, J., Richter, R., Valceanu, G., Otte, N., Teshima, M., Mirzoyan, R., Holl, P. and Koitsch, C., 2007, June. Development of back illuminated SiPM at the MPI semiconductor laboratory. In *Proceedings of International Workshop New Photon-Detectors* (pp. 1–7).

[24] Hu H, Wang Y, Liu P, Qin X, Fang J, Zhao H, Ma Z, Wei J (2022). Advanced back-illuminated silicon photomultipliers with surrounding P^+^ trench. IEEE Sens. J..

